# Occult mesenteric injury after blunt abdominal trauma

**DOI:** 10.1093/omcr/omaf083

**Published:** 2025-06-27

**Authors:** Jan Philipp Ramspott, Gaby Kass Abd Alahad, Hans-Joachim Meyer-Krahmer, Alexander D Bungert, Andreas Pascher, Reiner Schürmann

**Affiliations:** Department for General, Visceral and Transplant Surgery, University Hospital Münster, North Rhine-Westphalia, Waldeyerstraβe 1, 48149 Münster, Germany; Department for General and Visceral Surgery, Marienhospital Steinfurt, North Rhine-Westphalia, Mauritiusstraβe 5, 48565 Steinfurt, Germany; Department for Radiology, Marienhospital Steinfurt, North Rhine-Westphalia, Mauritiusstraβe 5, 48565 Steinfurt, Germany; Department for General, Visceral and Transplant Surgery, University Hospital Münster, North Rhine-Westphalia, Waldeyerstraβe 1, 48149 Münster, Germany; Department for General, Visceral and Transplant Surgery, University Hospital Münster, North Rhine-Westphalia, Waldeyerstraβe 1, 48149 Münster, Germany; Department for General and Visceral Surgery, Marienhospital Steinfurt, North Rhine-Westphalia, Mauritiusstraβe 5, 48565 Steinfurt, Germany

**Keywords:** abdominal trauma, mesenteric injury, bowel necrosis, peritonitis

A 28-year-old man was admitted to our emergency department following a frontal collision. He was a front-seat passenger, had his seat belt fastened, and was able to get out of the vehicle on his own. He reported pain in the right hemithorax, right arm, and both shoulders. His abdomen was soft with slight tenderness and seatbelt bruises in the right lower quadrant. The patient exhibited stable vital signs. Laboratory results showed leukocytosis (20 000/μl, normal 3900-10 900). A CT scan revealed a small amount of ascites but no acute hemorrhage or sign of solid organ injury (Fig. 1A). As a precaution, the patient was transferred to our intensive care unit for observation.

**Figure 1:**
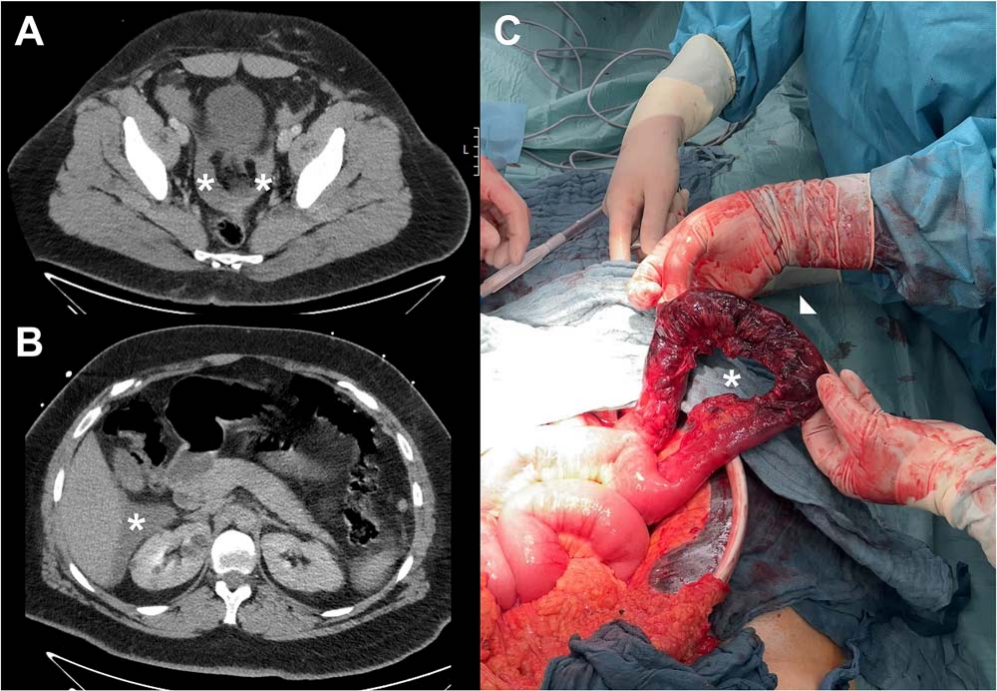
(A) Axial contrast-enhanced pelvic CT scan at patient’s admission and on (B) the second day post-trauma. Ascites is labeled by an asterisk. (C) Photograph (cranial-caudal orientation) of mesenteric injury (asterisk) with small bowel infarction (arrowhead).

Two days after the initial trauma he developed severe abdominal pain accompanied by peritonitis. An abdominal CT scan revealed increased ascites within the lesser pelvis and perihepatic region (Fig. 1B). Due to increased pressure within the abdominal compartment, an initial laparoscopic exploration was converted to laparotomy, which identified a mesenteric disruption of the terminal ileum, measuring 25 cm in length, complicated by small bowel infarction (Fig. 1C). Still no active hemorrhage was detected. Given the fact that the ischemia was located seven centimeters from the ileocecal valve, and due to the absence of fecal peritonitis, we carried out an ileocecal resection and an ileoascendostomy.

The postoperative course was uneventful, and the patient was discharged on the 10^th^ postoperative day. To facilitate early detection of a potential delayed anastomotic leak or demarcation of further bowel ischemia, rigorous clinical monitoring was advised.

Mesenteric injury following blunt abdominal trauma is rare, with an incidence of 0.3% [1]. In the absence of clear clinical or radiological signs, the diagnosis often remains elusive until the onset of peritonitis, resulting from bowel necrosis [2, 3]. Risk factors associated with mesenteric injury include abdominal inflammation, prior abdominal surgery, anemia, pelvic facture, and abdominal seatbelt evidence [4]. Current imaging modalities including contrast-enhanced CT scans and ultrasounds exhibit limited sensitivity [5]; that’s why the initial CT scan to detect mesenteric injury failed [6]. In case of severe fecal peritonitis, a damage control strategy with blind intestinal and temporary abdominal closure is recommended. In addition, early re-imaging and liberal surgical exploration are of pivotal concern to reduce high rates of morbidity and mortality [7].

## References

[1] Skelhorne-Gross G, Kenny J. Blunt and penetrating injury to the bowel: a review. Clin Colon Rectal Surg 2024;37:424–9. 10.1055/s-0043-1777668 39399140 PMC11466513

[2] Meissnitzer MW, Stättner S, Meissnitzer T. Small mesenteric hematoma following blunt abdominal trauma as early sign in computed tomography of occult small bowel perforation-report of 2 cases. Emerg Radiol 2014;21:647–50. 10.1007/s10140-014-1235-z 24832614

[3] Hamidian Jahromi A, Johnson L, Youssef AM. Delayed small bowel perforation following blunt abdominal trauma: a case report and review of the literature. Asian J Surg 2016;39:109–12. 10.1016/j.asjsur.2013.01.006 27016786

[4] Loftus TJ, Morrow ML, Lottenberg L. et al. Occult bowel injury after blunt abdominal trauma. Am J Surg 2019;218:266–70. 10.1016/j.amjsurg.2018.11.018 30509454 PMC6538466

[5] Granieri S, Altomare M, Bonomi A. et al. Contrast-enhanced CT scan (CECT) for the detection of hollow viscus and mesenteric injuries in blunt trauma - an updated systematic review of the literature and meta-analysis of diagnostic test accuracy. Eur J Trauma Emerg Surg 2024;50:2709–19. 10.1007/s00068-024-02667-9 39249527

[6] Murakami R, Tajima H, Kumazaki T. et al. CT findings of mesenteric injury after blunt trauma. CMIG Extra: Cases 2004;28:11–4. 10.1016/j.compmedimag.2003.09.005

[7] Bège T, Brunet C, Berdah SV. Hollow viscus injury due to blunt trauma: a review. J Visc Surg 2016;153:61–8. 10.1016/j.jviscsurg.2016.04.007 27209078

